# *In silico* protein structural analysis of PRMT5 and RUVBL1 mutations arising in human cancers

**DOI:** 10.1016/j.cancergen.2025.01.002

**Published:** 2025-01-17

**Authors:** Majd Al-Marrawi, Ruben C. Petreaca, Renee A. Bouley

**Affiliations:** a Neuroscience Undergraduate Program, The Ohio State University, USA; b Department of Molecular Genetics, The Ohio State University, Marion, USA; c Cancer Biology, The James Comprehensive Cancer Center, The Ohio State University, Columbus, USA; d Department of Chemistry and Biochemistry, The Ohio State University, Marion, USA

**Keywords:** Protein structural analysis, Mutation, Cancer genetics, Chromatin remodeling, DNA damage

## Abstract

DNA double strand breaks (DSBs) can be generated spontaneously during DNA replication and are repaired primarily by Homologous Recombination (HR). However, efficient repair requires chromatin remodeling to allow the recombination machinery access to the break. *TIP60* is a complex conserved from yeast to humans that is required for histone acetylation and modulation of HR activity at DSBs. Two enzymatic activities within the *TIP60* complex, *KAT5* (a histone acetyltransferase) and *RUVBL1* (an AAA+ ATPase) are required for efficient HR repair. Post-translational modification of *RUVBL1* by the *PRMT5* methyltransferase activates the complex acetyltransferase activity and facilitates error free HR repair. In *S. pombe* a direct interaction between *PRMT5* and the acetyltransferase subunit of the *TIP60* complex (*KAT5*) was also identified. The *TIP60* complex has been partially solved experimentally in both humans and *S. cerevisiae*, but not *S. pombe*. Here, we used *in silico* protein structure analysis to investigate structural conservation between *S. pombe* and human *PRMT5* and *RUVBL1*. We found that there is more similarity in structure conservation between *S. pombe* and human proteins than between *S. cerevisiae* and human. Next, we queried the COSMIC database to analyze how mutations occurring in human cancers affect the structure and function of these proteins. Artificial intelligence algorithms that predict how likely mutations are to promote cellular transformation and immortalization show that *RUVBL1* mutations should have a more drastic effect than *PRMT5*. Indeed, *in silico* protein structural analysis shows that *PRMT5* mutations are less likely to destabilize enzyme function. Conversely, most *RUVBL1* mutations occur in a region required for interaction with its partner (*RUVBL2*). These data suggests that cancer mutations could destabilize the *TIP60* complex. Sequence conservation analysis between *S. pombe* and humans shows that the residues identified in cancer cells are highly conserved, suggesting that this may be an essential process in eukaryotic DSB repair. These results shed light on mechanisms of DSB repair and also highlight how *S. pombe* remains a great model system for analyzing DSB repair processes that are tractable in human cells.

## Introduction

Eukaryotic cells wrap their DNA on histone nucleosomes creating a dynamic complex of DNA and protein known as chromatin [1]. Chromatin remodeling is the act of changing the interaction between proteins and DNA. It is an essential process for a variety of cellular functions including DNA packaging in the nucleus, development and differentiation, transcription, centromere and telomere establishment which maintain chromosome stability, chromosome segregation, regulation of repetitive “junk” DNA elements, replication, DNA damage repair, and many others [2–14]. Remodeling involves first “loosening” histone interaction with DNA primarily by acetylation, then removal of nucleosomes by specialized machineries [15–17]. A plethora of other histone modifications also facilitates effective chromatin remodeling. Modifications also serve as an epigenetic code (or histone code) that allows reversible association of other proteins with DNA [18–21].

Several chromatin remodeling machineries have evolved in eukaryotic cells, some specialized for unique processes and others with more pleiotropic functions [17,22–24]. The *TIP60* chromatin remodeler is a multi-subunit super-complex with histone acetyltransferase activities [25]. It has roles in transcription, global chromatin remodeling, and DNA double-strand break (DSB) repair [26–32]. An acetylase function resides in the *KAT5* enzyme, which is conserved throughout the eukaryotic domain [31,33,34]. The *TIP60* complex namesake originates from the initial identification of the histone acetyltransferase enzyme (only later also referred to as *KAT5* [35]) as an interactor and regulator of the human *HIV* Tat protein (*T*at *I*nteracting *P*rotein *60* kD) [36,37]. Subsequent analysis identified *TIP60/KAT5* as a subunit of a larger complex called NuA4 in the *Saccharomyces cerevisiae* yeast model system [38,39]. Structural analysis of the *TIP60*-complex in humans revealed that it can be separated into five submodules held together by the P400 scaffold, a *SWI/SNF* ATPase [40–45]. The *SWI/SNF* ATP-dependent chromatin remodeling complexes have pleiotropic functions and mutations have been identified in cancer cells [46]. A dynamic interplay between *KAT5* histone acetylation followed by *SWI/SNF* remodeling activity facilitates DSB repair [47].

The *KAT5* acetyltransferase activity evolved early in eukaryotes as it is present in both yeasts (*Saccharomyces cerevisiae* and *Schizosaccharomyces pombe*). In yeast, as in humans, it was directly linked to DNA DSB repair [26,48–50]. *KAT5* acetylates several residues of histone *H4* including H4K16, which promotes an open chromatin structure and facilitates long-range resection by allowing recruitment of specialized exonucleases and helicases [27,51–56]. Impeding *TIP60*-complex activity inhibits both long-range resection and homologous recombination (HR) mediated repair, indicating that it is required for efficient break repair [29,57].

In human cells, the functions of the *TIP60*-complex are modulated by the *PRMT5* arginine methyltransferase [58]. *PRMT5* methylates the *RUVBL1* subunit of the *TIP60*-complex at R205. *RUVBL1* is an AAA+ ATPase that participates in various chromatin remodeling functions [43, 44,59]. This *PRMT5* modification facilitates recruitment of the *TIP60*--complex to the breaks and promotes error-free repair HR repair [60]. *PRMT5* also regulates *KAT5* alternative splicing processes [61]. Dysregulation of *TIP60* DSB recruitment or effective splicing affects HR. The *RUVBL1-R205* residue is conserved in yeast, suggesting that this is an essential process in DNA damage repair, but remarkably, structures of the *S. cerevisiae* NuA4 complex did not identify Rvb1 (homologue of human *RUVBL1*) as a subunit [40,41,62]. However, a screen for *S. pombe* KAT5 (Mst1) interacting proteins identified the *PRMT5* (Skb1) but not the *RUVBL1* (Rvb1) homologue [48]. Subsequent analyses have shown that *PRMT5* is involved in recombination dependent chromatin remodeling [63]. Thus, *PRMT5* is a key regulator of DNA DSB repair but whether it has the same function in yeast (e.g. modulation of *TIP60*--complex activity) is debatable.

An answer to whether *PRMT5* also regulates *TIP60* in yeast is important because it suggests that the functions of the *TIP60* complex in modulating DSB repair is conserved throughout the eukaryotic domain. We and others have also shown that *KAT5* and *PRMT5* mutations are endemic in human cancers, which have destabilized DSB repair machineries [34,64–68]. Thus, answering this question is also important for understanding human disease.

In this report, we used *in silico* protein structure analysis to compare *PRMT5* and *RUVBL1* in humans and yeast. Additionally, we queried the COSMIC database [69], which catalogues mutations from analyzed or sequenced cancer genomes. We identified frequent and driver mutations and assessed their effects on the structures of *RUVBL1* and *PRMT5*. Our analysis shows a striking similarity between yeast and human species, highlighting the important function of these proteins in maintaining genome stability.

## Materials and methods

### COSMIC mutations

Files with COSMIC mutations for *PRMT5* and *RUVBL1* were downloaded on September 10, 2024. These data are for COSMIC version 100 released on 21 May 2024. For *PRMT5,* mutations were mapped on the ENST00000324366.12 transcript which codes for protein isoform *a*. while for *RUVBL1* the ENST00000322623.9 transcript was used which corresponds to protein isoform 1 as reported on NCBI (https://www.ncbi.nlm.nih.gov).

### CHASM analysis

The OPEN CRAVAT website (https://run.opencravat.org) [70] was used to extract p-values representing potential mutation driver status. We used the CHASM Plus tool [71] within this website which generates probability values and mutations with p-values below 0.05 were considered significant.

### Structural analysis

Experimental structures of the TIP60 complex (PDB ID: 8XVG) [44] and *PRMT5* structure (PDB ID: 4GQB) [72] were obtained from the Protein Data Bank. Mutated residues were mapped onto the respective structures using PyMOL version 3.0.3. The PDBePISA webserver was identify residues in protein-protein interfaces of the *TIP60*. Structure files were uploaded to the CUPSAT [73] web server to determine ΔΔG values and overall stability of each analyzed mutation. The PDBePISA tool (https://www.ebi.ac.uk/pdbe/pisa) was used to identify residues located in protein-protein interfaces.

### Structural homology analysis

The AlphaFold [74] models for human, *S. pombe*, and *S. cerevisiae PRMT5* and AlphaFold 3 [75] models of the *RUVBL1-RUVBL2* hexamer were generated. Alignments were performed in PyMOL using the align command with cycles set to 0 for global alignments, repeated with alignment tool, and the *R*oot *M*ean *S*quare *D*eviation (RMSD) values were collected, which indicate the similarity of the aligned structures. The lower the RMSD value, the higher the structural similarity of the homologs the default align command to allow for rejection of outliers.

All figures were created in Photoshop. Graphs and some statistical analyses were done in Excel or SPSS.

## Results and discussion

### Structural similarity of human and yeast homologs

The structure of human PRMT5 has been solved experimentally through X-ray crystallography bound to MEP50 and there are several co-crystal structures bound to various inhibitors [72,76,77]. MEP50 is a factor that enhances PRMT5 enzymatic activity [78,79]. The *S. pombe* and *S. cerevisiae* PRMT5 homologs do not have experimental structures available, thus AlphaFold was used to generate models [74]. The structure of human RUVBL1 has been successfully solved as a hexadimer with RUVBL2 [80] and in the TIP60 complex [44]. The *S. cerevisiae* RUVBL1 structure has also been solved and shown to form the same hexadimeric ring with RUVBL2 [81]. However, there are no experimental structures available of the *S. pombe* RUVBL1 and RUVBL2 homologs.

**Fig. 1.**
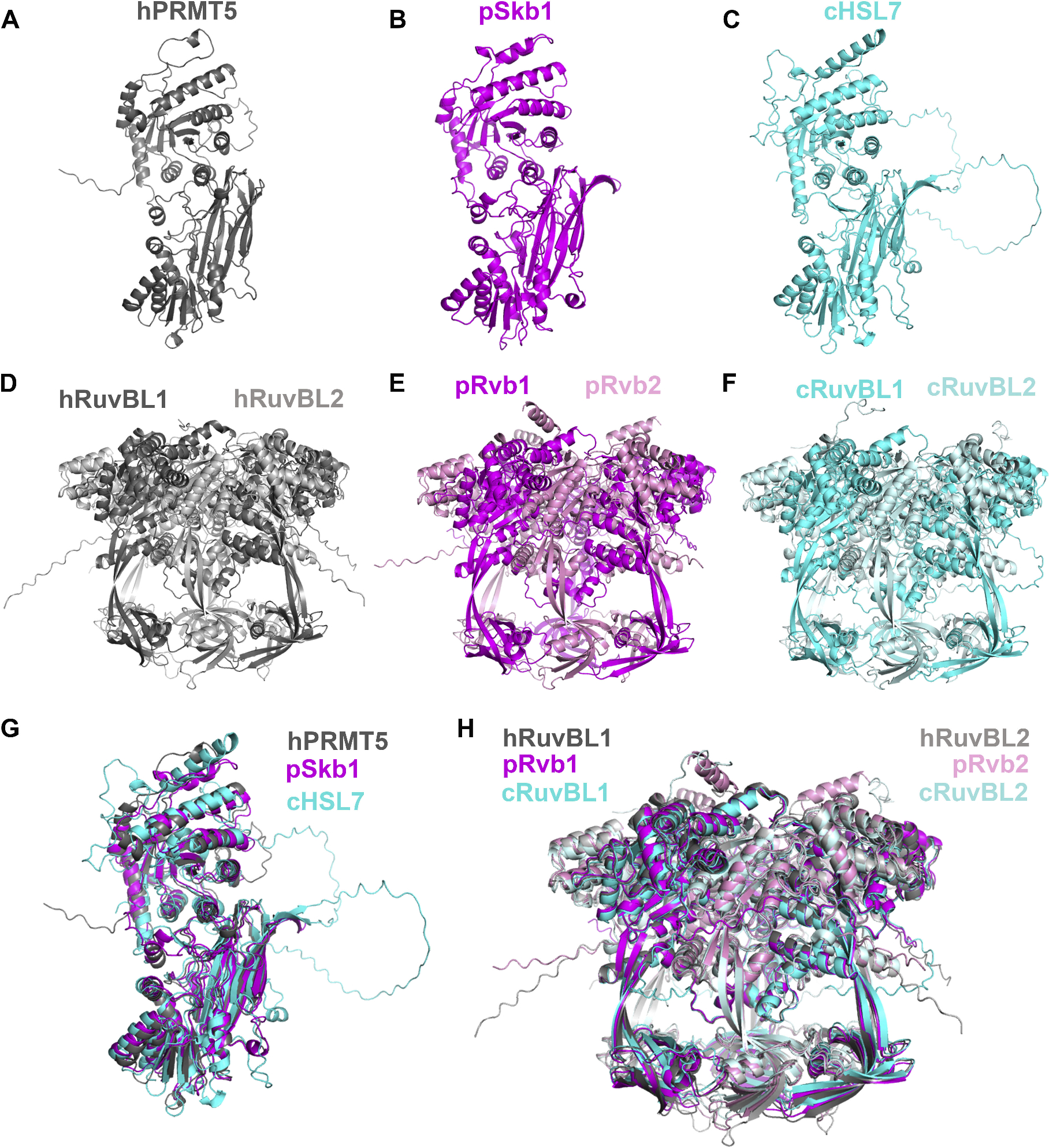
Alignments of human and yeast protein AlphaFold structures. AlphaFold structure models are shown for **A)** human PRMT5 in gray, **B**) *S. pombe* Skb1 in magenta, **C**) *S. cerevisiae* HSL7, **D**) human RUVBL1 in dark gray and RUVBL2 in light gray, **E**) *S. pombe* Rvb1 in magenta and Rvb2 in light magenta, **F**) *S. cerevisiae* RUVBL1 in cyan and RUVBL2 in light cyan. **G**) *S. pombe* Skb1 in magenta and *S. cerevisiae* HSL7 in cyan are aligned to human PRMT5 in gray. **H)** *S. pombe* Rvb1-Rvb2 in magenta (dark magenta for Rvb1 and light pink for Rvb2) and *S. cerevisiae* RUVBL1-RUVBL2 in cyan (dark cyan for RUVBL1 and light cyan for RUVBL2) are aligned to human RUVBL1-RUVBL2 in gray (dark gray for RUVBL1 and light gray for RUVBL2). Alignments and resulting RMSDs were calculated using PyMOL.

To perform alignments of the full-length proteins, AlphaFold 3 models were generated for the human, *S. pombe,* and *S. cerevisiae* protein structures (Fig. 1A–F). A visual inspection reveals that these proteins (PRMT5 and RUVBL1) look remarkably alike among the three species (human, *S. cerevisiae, S. pombe*). We next performed structural alignments to determine the similarity of these proteins among the three species (Fig. 1G & H). The global alignment of human PRMT5 and *S. pombe* Skb1 structures gave an RSMD of 4.64 Å (including all atoms), whereas with *S. cerevisiae* HSL7 the RMSD was 9.65 Å (Fig. 1G). Alignments were repeated in which outlier atoms were rejected during the alignment, giving RMSD values of 1.04 and 1.23 Å, respectively. The rejection of atoms during structural alignments in PyMOL allows for outliers to be excluded, which permits for more accuracy in dealing with regions of large flexibility. This demonstrates that the human and yeast proteins are very structurally similar and that the *S. pombe* protein is more similar to human than *S. cerevisiae*. RUVBL1 was similarly compared and AlphaFold 3 was used to generate models of the hexameric RUVBL1-RUVBL2 complex that has been shown experimentally (Fig. 1D–F). The overall alignment in PyMOL with no atoms rejected for the human and *S. pombe* structures was 36.41 Å (Fig. 1H). When the alignment was allowed to reject atoms during the alignment, a final RMSD of 1.22 Å was obtained. Similarly, the human and *S. cerevisiae* alignment gave an RMSD of 36.55 Å and when atoms were rejected, an RMSD of 1.29 Å (Fig. 1H). The visual comparison of these three homologs in Fig. 1 demonstrates how structurally similar these proteins are. Additionally, the *S. pombe* proteins were found to be more similar to the human proteins than *S. cerevisiae* analogs. These structural alignments demonstrate that interactions observed in *S. pombe*, such as that between Skb1/PRMT5 and Mst1/KAT5 [48], should be conserved in humans.

### Distribution of RUVBL1 and PRMT5 mutations in human cancers

We used the COSMIC database for this analysis. In a previous paper, we mapped all PRMT5 coding mutations and showed that they distribute throughout the entire region of the protein with no observable hotspots [66]. Additionally, we identified several truncating mutations that are likely to significantly destabilize the enzyme function. We undertook the same strategy to identify RUVBL1 mutations.

**Fig. 2.**
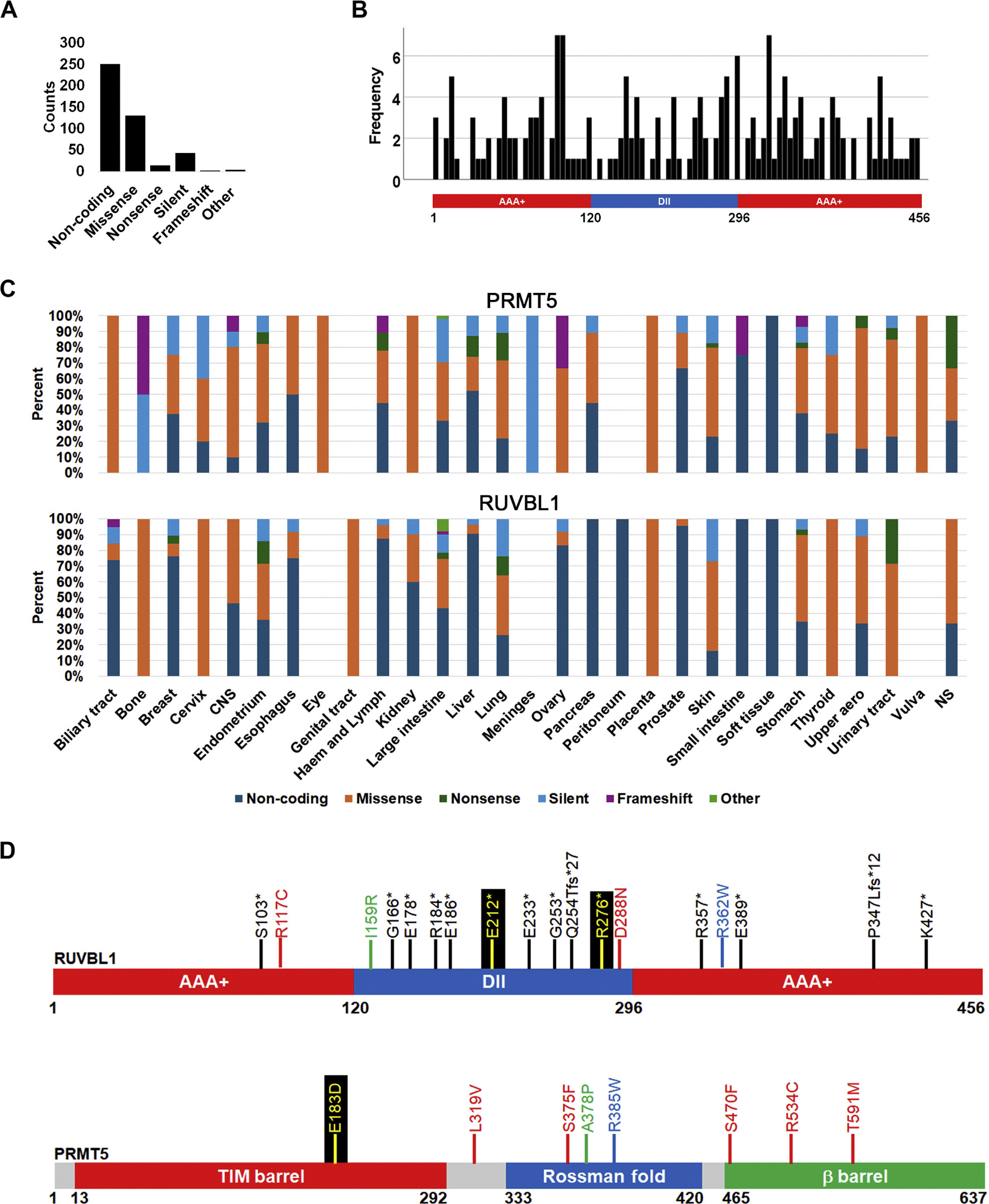
RUVBL1 COSMIC mutations. **A**. Distribution of all reported RUVBL1 mutations on COSMIC. **B**. RUVBL1 coding mutation density. A histogram of mutations was overlayed on a diagram of RUVBL1 adapted from [45,59]. **C**. Incidence of PRMT5 and RUVBL1 mutations in human cancers. **D**. RUVBL1 and PRMT5 mutations. The figure shows the position of RUVBL1 truncating mutations (black) and high frequency RUVBL1 and PRMT5 mutations (red). PRMT5 truncating mutations were reported previously [66]. Diagramed on the figure are also three pairs of co-occurring mutations (green, blue, yellow) that are predicted to affect the structure of RUVBL1 (please see text).

COSMIC reports both coding and non-coding (5′, 3′ UTR and intronic) mutations (Fig. 2A, Supplementary Table 1). We partitioned the coding mutations into missense, non-sense, silent, frameshift and other (complex). This distribution showed that most coding mutations are missense amino acid substitutions. Non-sense and frameshift mutations, which affect the protein function more drastically, were rarer. While most RUVBL1 mutations are non-coding, PRMT5 reported mutations are primarily coding (Supplementary Table 1) [66]. An analysis of the PRMT5 and RUVBL1 loci using the UC Santa Cruz genome browser (https://genome.ucsc.edu) reveals that RUVBL1 has longer introns than PRMT5. The PRMT5 genomic sequence is 8848 nucleotides long, of which 1914 (21.6 %) are coding. However, the RUVBL1 gene is 49,003 nucleotides and only 1371 (2.8 %) represent coding regions. Thus, the higher number of non-coding mutations in RUVBL1 is not unexpected.

Our previous analysis showed that PRMT5 mutations do not cluster in any protein region and there was no tendency for any domains of PRMT5 to be more affected than others [66]. We performed the same analysis for RUVBL1, which is characterized by two AAA+ domains at the N- and C-termini of the protein and a DII domain in the middle (Fig. 2B). The central region known as domain II (DII), which links the two AAA+ domains, contains an OB (oligonucleotide binding) fold region. Crystal structure analysis shows that the two AAA+ domains interact with each other while DII juts out of the structure [82]. A histogram plot of all RUVBL1 mutations reported on COSMIC indicates that there is no tendency for mutations to accumulate in any of the three domains (Kolmogorov-Smirnov test for uniformity p-value = 0.645) (Fig. 2B).

### Analysis of frequent RUVBL1 and PRMT5 mutations appearing in cancer cells

PRMT5 and RUVBL1 mutations are detected in every cancer (Fig. 2C). This is not unexpected since these two genes are involved in maintaining genome stability, a process destabilized in all cancers. We next analyzed the potential effect of mutations on the function of these proteins. Several recent analyses of cancer genomes have suggested that when querying large databases, mutations occurring in 4 to 5 samples or more should be interpreted as frequent [83–85]. We identified three PRMT5 (S375F, R534C/H, T591M) and one RUVBL1 (R117C) frequent mutations in the COSMIC dataset (Fig. 2D, Table 1). Two mutations occurring in only three samples in PRMT5 (L319V, S470F) and one in RUVBL1 (D288N) were also analyzed. To understand the impact of these mutations, we performed two tests: CHASM and CUPSAT [71,73]. CHASM is an artificial intelligence algorithm that classifies single amino acid substitutions as either driver or passenger. Driver mutations are more likely to contribute to cellular transformation and immortalization than passenger mutations [86]. The algorithm generates a probability value, and we interpreted all mutations with a p-value below 0.05 to be driver. CUPSAT was used to predict if mutations in PRMT5 or RUVBL1 would impact the stability of the protein structure. The ΔΔG value provided compares the ΔG of protein folding for the WT and mutant proteins to predict whether a mutation would destabilize (negative ΔΔG) or stabilize (positive ΔΔG) the protein structure. The larger the value of the ΔΔG, the greater affect it has on the protein folding and structure.

Our previous analysis of PRMT5 did not identify any mutations with a CHASM p-value below 0.05 [66] suggesting that no substitution is likely to drastically affect its function. Consequently, none of the frequent mutations identified here are classified as driver (Table 1). Three PRMT5 mutations (L319V, S470F, and T591M) were predicted to significantly destabilize the protein structure with CUPSAT ΔΔG values less than −2.0 kcal/mol. One frequent PRMT5 mutation (S375F) was predicted to significantly stabilize the protein structure with a ΔΔG value greater than +2.0 kcal/mol. For RUVBL1, neither of the frequent mutations (R117C or D288) were predicted to significantly impact protein structural stability.

We also identified several RUVBL1 truncating mutations (Fig. 2D). Truncating mutations have a more drastic effect on gene function (often inactivating) because they can delete entire domains. Remarkably, most truncating mutations occur in the DII domain, in effect, eliminating the second AAA+ domain. This DII domain contains the R205 residue that PRMT5 has been shown to methylate, therefore, these truncations would affect this interaction. Our previous analysis of PRMT5 mutations showed that there is a higher probability for them occurring in the N-terminus of the protein and results in deletion of nearly the entire protein sequence [66]. Thus, PRMT5 truncating mutations are expected to completely inactivate the function of the gene.

### Analysis of co-occurring PRMT5 and RUVBL1 mutations

Given the functional interaction between PRMT5 and RUVBL1, we wanted to analyze co-mutations between these two genes in human cancers. The occurrence of co-mutations between two genes suggests that they function in the same pathway while mutual exclusivity suggest that they function in two parallel pathways and inactivating one pathway may render the second pathway essential for viability.

We identified multiple samples with co-occurring mutations in various human cancers (Table 1). As expected, none of the PRMT5 co-occurring mutations have a statistically significant CHASM value, but ten out of eighteen RUVBL1 substitutions are predicted to have a driver effect (CHASM p value below 0.05) (Table 1). This suggests that destabilization of RUVBL1 has a greater effect on cellular transformation than PRMT5. CUPSAT analysis revealed that two co-occurring PRMT5 mutations were significantly destabilizing (G7W and A378P) and two were stabilizing (G145C and S375F). In fact, only one of all the PRMT5 frequent or co-occurring mutations was predicted to not significantly impact protein structure (R534C). Most of the co-occurring RUVBL1 mutations did not have large enough ΔΔG values to have a significant effect. However, there were two co-occurring mutations, I159R and R362W, that were predicted to significantly destabilize the protein structure (Fig. 2D, Table 1). One PRMT5 mutation (E183D) co-occurred with two RUVBL1 truncations (E212*, R276*) (Fig. 1D, Table 1). Although the RUVBL1 truncations are almost certainly inactivating, structural analysis shows that the PRMT5 mutation is not present near the active site, but CUPSAT predicts this mutation to be destabilizing to the overall structure.

This analysis of co-occurring mutations suggests that although mutations between PRMT5 and RUVBL1 do co-occur, it is rare that the functions of both enzymes are significantly destabilized in the same sample. The genetic interpretation of these data is that although PRMT5 does modulate the function of RUVBL1 and TIP60 complex (e.g. working in the same pathway), they have other functions essential for cellular viability (e.g. different pathways). Indeed, both PRMT5 and RUVBL1 have pleiotropic functions not related to DSB repair [59,67,87].

### Structural analysis of RUVBL1 and PRMT5 mutated residues

**Fig. 3.**
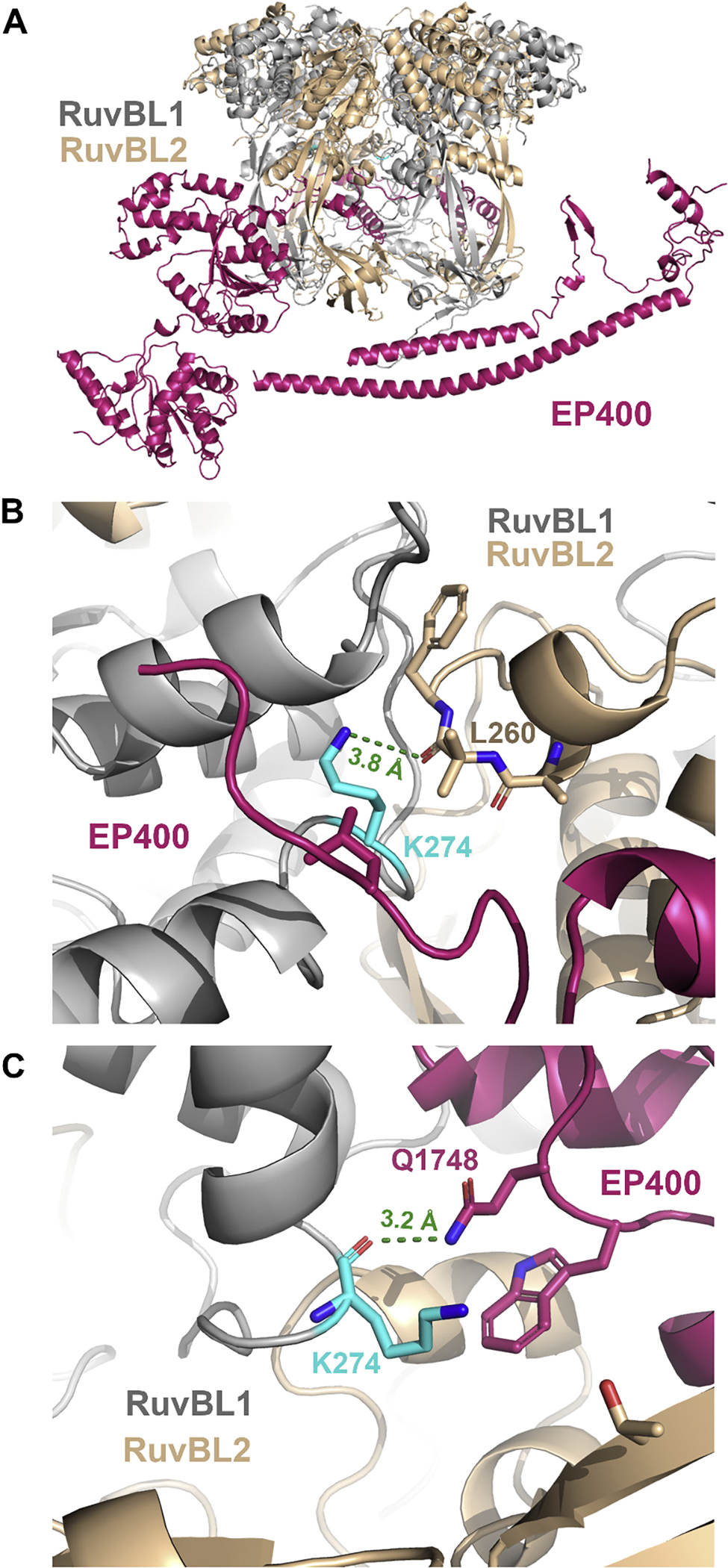
The K274 residue found in the RUVBL1 structure interacts with both RUVBL2 and EP400. **A)** An overview of the human RUVBL1-RUVBL2 hexamer interacting with EP400 from PDB 8XVG. The RUVBL1 subunits are shown in silver, the RUVBL2 subunits in gold, and the EP400 protein is shown in dark pink. **B)** The K274 residue of RUVBL1 (silver) is shown in cyan sticks forming a hydrogen bond (green dashed line) with L260 of RUVBL2 (gold). **C)** The K274 residue of RUVBL1 (silver) is shown in cyan sticks forming a hydrogen bond (green dashed line) with Q1748 of EP400 (dark pink).

The recent cryo-EM structure of TIP60 (PDB ID: 8XVG) was used to identify RUVBL1 protein-protein interactions using PDBePISA. The two frequent mutations (R117 and D288) were both found in the interface between RUVBL1 and RUVBL2. Of the 15 RUVBL1 mutated residues co-occurring with PRMT5 (Table 1), 10 were found to be in the interface between RUVBL1 and RUVBL2: A16, A62, P95, R117, K274, V337, R362, R378, P412, and A440. In addition, two of the co-occurring mutated residues were found in the interface between RUVBL1 and EP400: A199, K274. EP400 belongs to the INO80 family and was shown in a previous cryo-EM structure to serve as a central scaffold for the other subunits of the TIP60 complex [44]. This structure also showed that a portion of EP400 between lobes 2 and 3 inserts into the RUVBL1-RUVBL2 hexamer forming numerous interactions. Our results show that K274 is important for the both formation of the RUVBL1-RUVBL2 hexameric ring as well as the interaction with EP400 to anchor it to the rest of the TIP60 complex (Fig. 3). Thus, the majority of the identified frequent or co-occurring mutations are found in residues in a protein-protein interface. None of the frequent or co-occurring PRMT5 mutations were found to be directly within the active site or the interface with MEP50. However, S375 is located near the active site pocket and mutation to a bulky phenylalanine could cause a shift in the helix it is located on, which would affect the active site pocket.

### Analysis of conservation in yeast

The PyMOL alignments in Fig. 1 were used to identify the analogous residues in *S. pombe* to the human residues that were found to be either frequent or co-occurring mutations in COSMIC (Table 1). The majority of the RUVBL1 mutated residues were conserved between the species, with only 1 residue that was identified as non-conserved (V337) of the 16 total mutated residues. For PRMT5, 3 of the 8 mutated residues were not conserved between human and *S. pombe:* S375, G7, and G145.

## Conclusion

The structural analyses presented in this paper suggest that mutations in *PRMT5* seem to select for those that significantly affect the overall stability of the protein, but do not directly affect the active site or interactions with MEP50. *RUVBL1* mutations appear to be selected for residues that are involved in protein-protein interactions and the mutations generally do not affect overall protein stability.

This study also highlights the significance of these proteins in genome stability in eukaryotes. Previous structural analyses have shown that there is high conservation of these proteins between *S. cerevisiae* and humans. We show here through *in silico* structural analysis that both PRMT5 and RUVBL1 structures are also highly conserved in *S. pombe*. Therefore, the yeasts remain excellent model systems for studying basic mechanisms of DNA damage repair which are tractable in human cancers.

We are cognizant of the fact that the predictions made here would need to be validated by experimentation and this poses a limitation to the interpretation of these results. Nevertheless, these studies should inform experiments in both yeast and human cancer genetics.

## Supplementary Material

Supplementary Table 1

Supplementary material associated with this article can be found, in the online version, at doi:10.1016/j.cancergen.2025.01.002.

## Figures and Tables

**Table 1:** Analysis of RUVBL1 and PRMT5 mutations.

PRMT5					RUVBL1						
*Human mutation* ^1^	*S. pombe residue* ^2^	*CHASM p-value* ^3^	*Predicted ΔΔG (kcal/mol)* ^4^	*Overall stability* ^5^	*Human mutation* ^1^	*S. pombe residue* ^2^	*CHASM p-value* ^3^	*Predicted ΔΔG (kcal/mol)* ^4^	*Overall stability* ^5^	*Cancer type*	*Other remarks*
*Frequent*											
L319V	L311	0.07	−2.63	Dest.						Urinary tract	
S375F^6^	A362	0.14	6.83	Stab.						Skin	
S470F	S458	0.01	−3.88	Dest.						Skin, lung	
R534C/H^7^	R527	0.166	0.32	Stab.						Large intestine, skin, endometrium, stomach	
T591M	Y587	0.30	−2.61	Dest.						Stomach, large intestine	
					R117C^6^	R118	0.0164	1.17	Stab.	Large intestine, skin, stomach	
					D288N	E289	0.030	−0.25	Dest.	Large intestine, prostate	
*Co-occurring*											
G7W	R7	0.195	−2.29	Dest.	K274N	K275	0.0348	−0.22	Dest.	Skin	Female, 49y
G145C	T154	0.0963	2.17	Stab.	R378L	R379	0.0649	0.47	Stab.	Thyroid	Male, 44Y
S375F	A362	0.14	6.83	Stab.	P412Q	P413	0.064	1.0	Stab.	Skin	Mai, 55y
A378P	A365	0.0705	−2.3	Dest.	I159R	L160	0.0128	−3.25	Dest.	Lung	Male, 48y
D531N	H524	0.306	−0.08	Dest.	A16T	I16	0.164	−1.23	Dest.	Stomach	Male, 68y
R534H	R527	0.197	−0.08	Dest.							
R220H	R211	0.136	5.88	Stab.	A62T	G63	0.0471	1.93	Stab.	CNS	N/A
S375F	A362	0.14	6.83	Stab.	P92L	P93	0.0122	−0.82	Dest.	Skin	N/A
G466V	S454	0.192	0.98	Stab.	P95Q	P96	0.0213	1.41	Stab.	Placenta	Female, 39Y
G466R	S454	0.223	−0.22	Dest.	R117C	R118	0.0164	1.17	Stab.	Large intestine	Male, 71y
F277L	S269	0.124	−2.49	Dest.	A119T	A120	0.017	−1.52	Dest.	Stomach	Female, 78y
W89*	W96	N/A	N/A	N/A	A199V	A200	0.0313	−0.49	Dest.	Skin	Female, 58y
R256W	L247	0.149	2.75	Stab.	V337A	T338	0.0513	0.32	Stab.	Large intestine	Cell line
R385W	T372	0.333	−1.5	Dest.	R362W	R363	0.0513	−4.42	Dest.	CNS	N/A, 6.8y
R91H	E98	0.185	−1.4	Dest.	Q369H	S370	0.0505	−0.36	Dest.	Endometrium	Female, 53y
L253F	I244	0.149	−0.34	Dest.	A440V	A441	0.0446	−0.08	Dest.	CNS	N/A, 12.5y
P541S	Q534	0.266	−3.11	Dest.	A199V	A200	0.0313	−0.49	Dest.	Skin	N/A, N/A
E183D	-	0.266	−1.42	Dest.	E212*		N/A	N/A		Breast	Female, 68y
					R276*		N/A	N/A			
S87Y	S94		−4.78	Dest.	E233*		N/A	N/A		Endometrium	Female, 55y

1 As reported on COSMIC.

2 Based on alignment of the two sequences.

3 Value generated using the OPEN CRAVAT CHASMPlus algorithm.

4 The value is given for the human mutation.

5 The interpretation is for the human mutation. Dest. = destabilizing; Stab. = stabilizing.

6 This mutation is both high frequency and co-occurring.

7 A different co-occurring residue is substituted at this position.

## Data Availability

All data are available on public repositories COSMIC.
